# 

**Published:** 1890-12

**Authors:** 


10 cts. a copy. DECEMBER, 1890. $1.00 per year.

### HALL'S JOURNAL OF HEALTH

ESTABLISHED 1854

CONTENTS.

Vol. 37. No. 12.

Headquarters—218 to 222 Fulton St., near Greenwich. Office, Room 18.

Entered as Second-Class Matter at the Post-Office, New York.

